# Carboxylesterase Notum Is a Druggable Target to Modulate Wnt Signaling

**DOI:** 10.1021/acs.jmedchem.0c01974

**Published:** 2021-03-30

**Authors:** Elliott D. Bayle, Fredrik Svensson, Benjamin N. Atkinson, David Steadman, Nicky J. Willis, Hannah L. Woodward, Paul Whiting, Jean-Paul Vincent, Paul V. Fish

**Affiliations:** † Alzheimer’s Research UK UCL Drug Discovery Institute, University College London, Cruciform Building, Gower Street, London WC1E 6BT, U.K.; ‡ The Francis Crick Institute, 1 Midland Road, Kings Cross, London NW1 1AT, U.K.

## Abstract

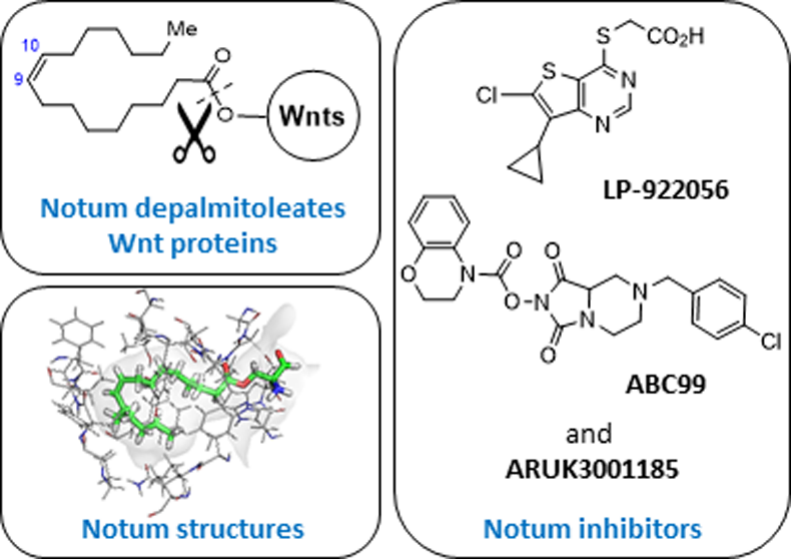

Regulation of the Wnt signaling pathway is critically important for a number of cellular processes in both development and adult mammalian biology. This Perspective will provide a summary of current and emerging therapeutic opportunities in modulating Wnt signaling, especially through inhibition of Notum carboxylesterase activity. Notum was recently shown to act as a negative regulator of Wnt signaling through the removal of an essential palmitoleate group. Inhibition of Notum activity may represent a new approach to treat disease where aberrant Notum activity has been identified as the underlying cause. Reliable screening technologies are available to identify inhibitors of Notum, and structural studies are accelerating the discovery of new inhibitors. A selection of these hits have been optimized to give fit-for-purpose small molecule inhibitors of Notum. Three noteworthy examples are LP-922056 (**26**), ABC99 (**27**), and ARUK3001185 (**28**), which are complementary chemical tools for exploring the role of Notum in Wnt signaling.

## Introduction to Wnt Signaling

**Figure 1:**
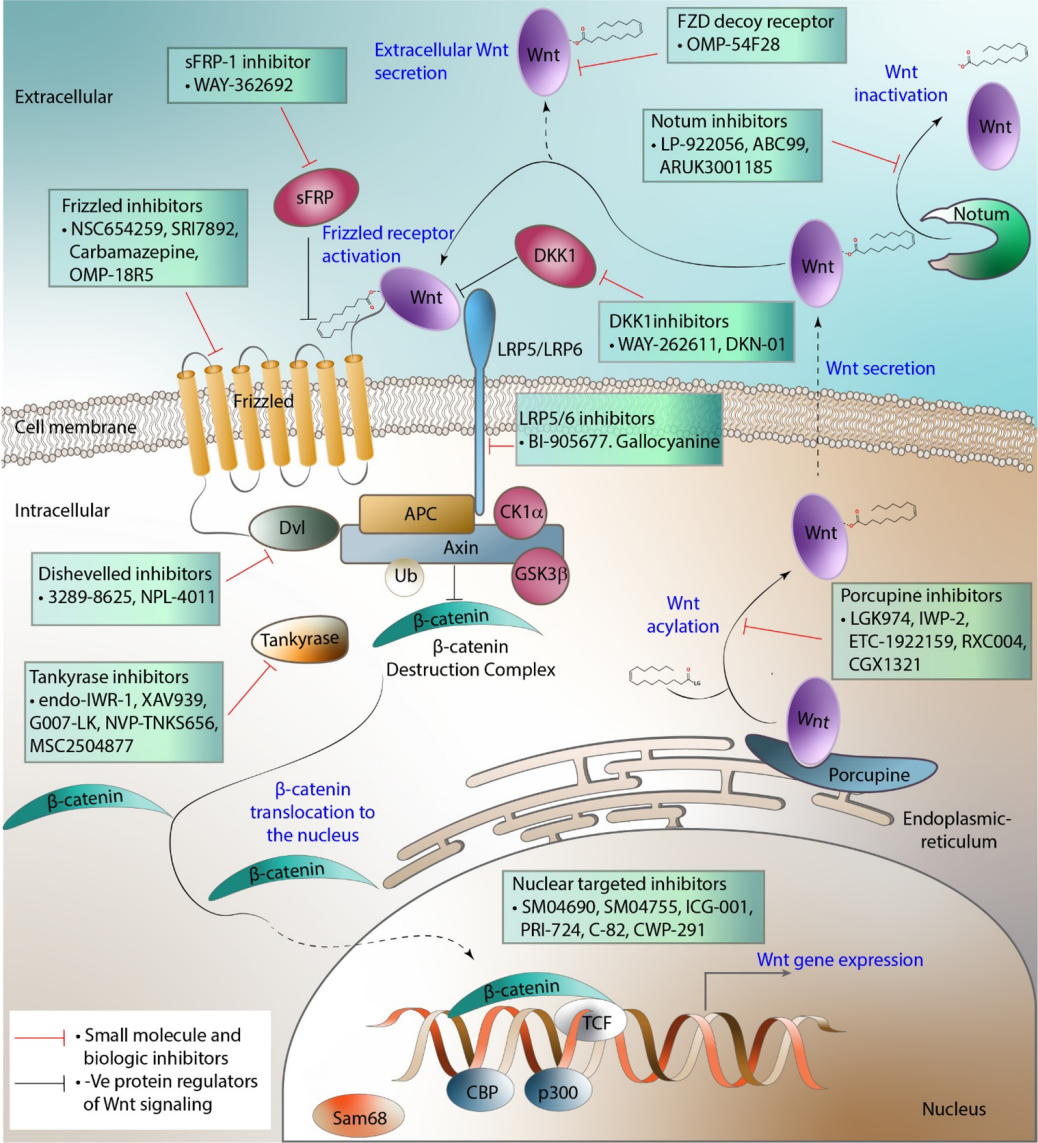
Overview of the canonical Wnt signaling pathway.

During development and adult homeostasis, cells use secreted proteins to communicate and coordinate their activities. Most of the known signaling proteins fall into a relatively small number of families. One such family encompasses the Wnt proteins (pronounced “wint”), which were discovered independently by developmental biologists and cancer biologists. Indeed, the name Wnt is a fusion of *wingless*, a gene required for *Drosophila* wing specification^1^ and embryo patterning,^2^ with int-1, a viral integration site associated with mammary tumors.^3^ Thus, from the start, Wnt genes embody the molecular links between normal development and tumorigenesis. Since their original identification, Wnt proteins have been found across metazoans ranging from worms to humans.^4^ Subsequent work to identify all the key signal transduction components further highlighted the highly conserved nature of the Wnt signaling pathway. Canonical signaling is initiated by binding of a Wnt to a member of the Frizzled (FZD) family of receptor, leading to recruitment of the co-receptor LRP5/6^5^ and inactivation of a protein complex that normally degrades β-catenin.^6^ Thus, in the presence of Wnt, β-catenin accumulates and, along with a number of cofactors,^7^ triggers the transcriptional activation of target genes. Wnt also activates noncanonical signaling, for example, the planar cell polarity pathway, which is also initiated by binding to a FZD receptor but does not involve LRP5/6 and β-catenin.^8^ In a minor pathway, Wnts can also exert their influence independently of FZD receptors, via other receptors such as ROR or RYK.^9^ Here we focus on FZD-dependent Wnt signaling, which accounts for most of Wnt proteins’ activities (Figure 1).

A characteristic feature of Wnts (including all 19 human Wnts) is that they are post-translationally appended with a palmitoleate moiety.^10,11^ Without this lipid, the affinity of Wnts for FZD is greatly reduced, an observation that can be readily understood from structural studies, which showed that the Wnt lipid establishes close contact with a hydrophobic groove present in the extracellular domain of FZD.^12^ As expected therefore, Wnt mutant proteins lacking the conserved serine that is normally palmitoleoylated display low activity *in vivo*.^13,14^ Likewise, genetic abrogation of Porcupine, a membrane-bound *O*-acyl transferase (MBOAT) solely devoted to Wnt palmitoleoylation, phenocopies inactivation of all Wnts.^11,15,16^ The same effect is achieved by chemical inhibitors of Porcupine,^17^ which are currently undergoing clinical trials for the treatment of cancers caused by Wnt overexpression. Thus, classical and chemical genetic approaches have highlighted the importance of the Wnt lipid.

Loss of function studies have shown that Wnt proteins control a wide range of physiological functions such as vertebrate axis specification, growth control, bone regeneration, synaptogenesis, stem cell maintenance, and many more.^18−20^ Conversely, excess or ectopic Wnt signaling has been associated with various diseases, including cancer, vascular disease, Alzheimer’s disease, and developmental defects. Most prominently, overactivation of Wnt signaling is thought to account for the majority of colorectal cancers and to be a key contributor to many other cancers.^21^ It is clear therefore that Wnt signaling needs to be finely balanced in time and space such that it is high enough to control essential functions such as stem cell maintenance, while avoiding the deleterious effects of excess signaling. This is achieved by an array of feedback mechanisms that modulate the spread of Wnt ligands, their activity, receptor availability, or the function of intracellular signal transduction components.^22^ Here, we consider one such mechanism, that mediated by the secreted protein Notum.

This Perspective will provide a summary of current and emerging therapeutic opportunities in modulating Wnt signaling, especially through inhibition of Notum carboxylesterase activity. Notum is emerging as a druggable target to modulate Wnt signaling. Our emphasis will be on structural biology, modern hit finding approaches, and medicinal chemistry strategies to deliver fit-for-purpose chemical tools. Three noteworthy examples of inhibitors of Notum activity are LP-922056 (**26**), ABC99 (**27**), and ARUK3001185 (**28**), which are complementary chemical tools for exploring the role of Notum in Wnt signaling.

## Wnt Pathway and Determination of Notum Function

Notum was identified independently by two groups studying the regulation of Wingless signaling in *Drosophila*. In the group of Gerlitz and Basler, an enhancer trap screen for genes that are activated by Wingless signaling led to the identification of a mutation that caused an expansion of presumptive wing tissue.^23^ The corresponding gene was hence named *wingful*. At the same time, Giraldez et al. found that overexpression of what turned out to be the same gene caused the opposite phenotype, the loss of wing tissue,^24^ as well as enlargement of the notum, an anatomical structure at the back of the fly. The latter phenotype led the authors to name the gene *notum*, a name that somehow superseded *wingful*. Both studies showed that Notum is a target of Wingless signaling and that its protein product is a potent inhibitor of Wingless signaling. In *Drosophila*, Notum is probably a universal feedback inhibitor since its expression is activated at all known locations of Wingless signaling.^25^ In mice, Notum is also expressed at many,^26^ though not all, sites of Wnt signaling, including in β-catenin-driven tumors.^27^ Therefore, in vertebrates as in flies, Notum is a feedback inhibitor that contributes to dampening Wnt signaling.

**Figure 2:**
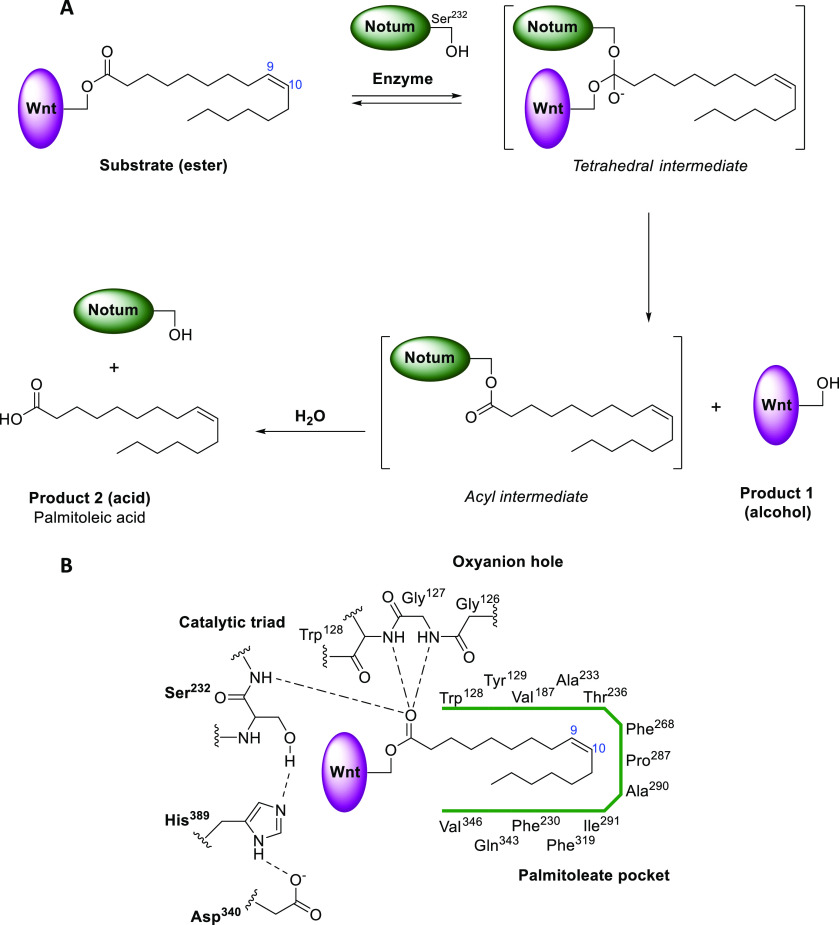
(A) Chemical reaction of the carboxylesterase activity of Notum acting on Wnt to remove the palmitoleate group. (B) Two-dimensional schematic of binding interactions. The Gly^127^–Trp^128^ amide participates in formation of the oxyanion hole in addition to the canonical Ser^232^–Ala^233^ and Gly^126^–Gly^127^ amides. Catalytic triad (Ser^232^, His^389^, Asp^340^) in bold.

How does Notum inhibit Wnt signaling? The presence of a signal peptide indicated at the outset that Notum acts in the extracellular space. Protein sequence similarity between Notum and plant pectin acetylesterases suggested that Notum could modify the glycosaminoglycans of glypicans, glycosylphosphatidylinositol (GPI)-anchored proteoglycans to which Wnts and other growth factors are known to bind.^24^ However, subsequent biochemical experiments cast doubt on this model and suggested instead that Notum could be a phospholipase that cleaves the GPI anchor of glypicans thus releasing them from the cell surface, along with any bound Wnt.^28^ If the molecular target of Notum was really a glypican, one would expect more pleiotropic effects since a number of extracellular proteins besides Wnts bind to glypicans (e.g., Hedgehog, FGF, BMP). Yet, genetic analysis showed that Notum is a Wnt-specific inhibitor.^29^ The phospholipase model could be unambiguously excluded by structural analysis and enzymatic assays, which showed that Notum is a carboxylesterase (Figure 2).^29^

The crystal structure of Notum also revealed a hydrophobic pocket that could accommodate *cis*-palmitoleate, an observation that naturally led to the hypothesis that Notum could hydrolyze the O-linkage of palmitoleate to Wnts. This was indeed verified *in vitro* by MALDI analysis of palmitoleoylated peptides treated with recombinant Notum. Interestingly, structural analysis did uncover several heparin binding sites at the surface of Notum. Such sites not only account for Notum’s ability to bind glypicans, they also provide a molecular framework for the documented genetic interactions between Notum and glypicans. It is now accepted that Notum is a glypican-dependent Wnt deacylase.^29^

## Small Molecule Approaches to Modulate Wnt Signaling

The strong links between aberrant Wnt signaling and multiple human diseases has promoted significant interest in the Wnt pathway machinery as targets for drug therapies. To date, most efforts have been directed at inhibiting Wnt signaling, in particular signaling through the canonical pathway and ultimately β-catenin regulated gene transcription.^30^ The most appropriate target(s) for intervention in the Wnt signaling cascade has been the topic of much debate as there needs to be clear therapeutic benefit (efficacy) with a suitable safety index.^31,32^

**Figure 3:**
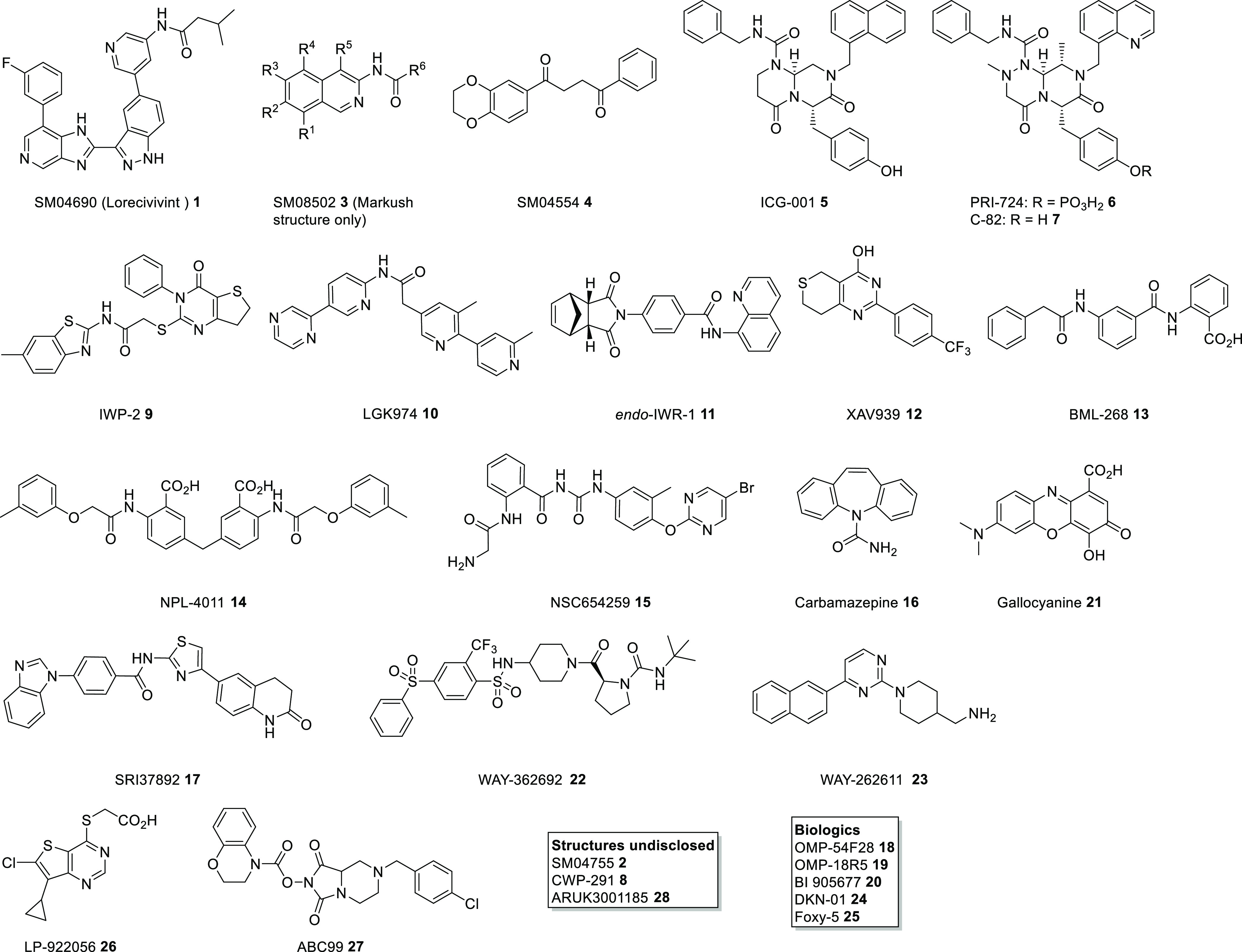
Chemical structures of selected small molecules modulating the Wnt pathway.

A number of recent reviews have summarized the state of the art with regard to Wnt inhibition with a focus on cancer therapeutics.^33−36^ As such, discussion in this section will be limited to Wnt pathway inhibitors either that are in clinical trials or for which there is structural information on the ligand–protein interaction. Small molecule approaches toward activating or enhancing Wnt signaling are also described. The application of biological agents that modulate the Wnt pathway that have progressed to clinical trials will be briefly discussed for comparison (Tables 1 and 2; Figure 3).

**Table 1:** Selected Small Molecule Tools Targeting Wnt Pathway Components

compound identifier	molecular target	effect on canonical Wnt signaling	structural information in public domain	furthest progress to date	refs
ICG-001, **5**	CBP	inhibit	no	preclinical	(45)
IWP-2, **9**	Porcupine	inhibit	no	preclinical	(51)
endo-IWR-1, **11**	TNKS1/2	inhibit	yes	preclinical	(51)
XAV939, **12**	TNKS1/2	inhibit	yes	preclinical	(57,59)
G007-LK	TNKS1/2	inhibit	yes	preclinical	(60,63)
NVP-TNKS656	TNKS1/2	inhibit	no	preclinical	(61)
MSC2504877	TNKS1/2	inhibit	no	preclinical	(62)
BML-268, **13**	DVL3	inhibit	yes	early discovery	(65)
NPL-4011, **14**	DVL3	inhibit	yes	early discovery	(66)
NSC654259, **15**	FZD8	inhibit	yes	early discovery	(67)
carbamazepine, **16**	FZD8	inhibit	yes	preclinical^a^	(68)
SRI37892, **17**	FZD7	inhibit	yes	early discovery	(70)
gallocyanine, **21**	LRP6	enhance	yes	early discovery	(77)
WAY-362692, **22**	sFRP-1	enhance	no	preclinical	(82−84)
WAY-262611, **23**	DKK1	enhance	no	preclinical	(85)
LP-922056, **26**	Notum	enhance	yes	preclinical	(95)
ABC99, **27**	Notum	enhance	no	preclinical	(118)
ARUK3001185, **28**	Notum	enhance	no	preclinical	(126)

a Carbamazepine is an approved anticonvulsant drug used clinically in the treatment of epilepsy and neuropathic pain.

**Table 2:** Summary of Agents in Clinical Trials

compound identifier	molecular target	clinical phase	NCT reference	indications
SM04690, **1**	CLK2/DYRK1A	phase 2	NCT03727022	knee osteoarthritis
		phase 2	NCT03706521	knee osteoarthritis
SM04755, **2**	CLK2/DYRK1A	phase 2	NCT03229291	tendinopathy
		phase 1	NCT02191761	colorectal, gastric, hepatic and pancreatic cancer
		phase 1	NCT03502434	tendinopathy
SM08502, **3**	pan-CLKs/DYRK1A	phase 1	NCT03355066	advanced solid tumors
SM04554, **4**	not reported	phase 2/3	NCT03742518	androgenetic alopecia
		phase 2	NCT02503137	androgenetic alopecia
		phase 2	NCT02275351	androgenetic alopecia
PRI-724, **6**	CBP	phase 2	NCT03620474	hepatitis b and c, liver cirrhosis
			NCT04047160	primary biliary cholangitis, liver cirrhosis
C-82, **7**	CBP	phase 2	NCT02349009	systemic scleroderma
			NCT02432027	psoriasis
CWP-291, **8**	Sam68	phase 1/2	NCT03055286	acute myeloid leukemia
		phase 1	NCT02426723	multiple myeloma
		phase 1	NCT01398462	acute myeloid leukemia, myelofibrosis
LGK974, **10**	Porcupine	phase 1	NCT02278133	metastatic colorectal cancer
		phase 1	NCT01351103	multiple solid malignancies
		phase 2	NCT02649530	squamous cell carcinoma (withdrawn)
OMP-54F28, **18**	Wnts	phase 1	NCT02069145	liver cancer
		phase 1	NCT02092363	ovarian cancer
		phase 1	NCT02050178	pancreatic cancer
		phase 1	NCT01608867	solid tumors
OMP-18R5, **19**	FZD 1,2,5,7,8	phase 1	NCT01345201	solid tumors
		phase 1	NCT02005315	pancreatic cancer
		phase 1	NCT01957007	solid tumors
		phase 1	NCT01973309	metastatic breast cancer
BI-905677, **20**	LRP5/LRP6	phase 1	NCT03604445	solid tumors
DKN-01, **24**	DKK1	phase 2	NCT03645980	liver cancer
		phase 2	NCT04166721	metastatic esophageal and gastric cancer
		phase 2	NCT03837353	prostate cancer
		phase 2	NCT03395080	gynecological cancers
Foxy-5, **25**	Wnt5a	phase 1	NCT02655952	metastatic breast, colon and prostate cancer
		phase 2	NCT03883802	colon cancer

### Wnt Pathway Inhibitors and Activators

A popular approach for investigating small molecule mediated modulation of Wnt activity has been through cell-based functional assays, such as TOP-flash, in which cells transiently or stably express luciferase proteins under the TCF/LEF promoter.^37^ A large number of compounds have been reported to inhibit Wnt signaling using this approach, in particular natural products,^38^ many without apparent follow-up or target identification. Compounds of interest without disclosed molecular targets will be discussed here. A number of compounds that modify Wnt signaling have been disclosed by Samumed LLC and are currently in clinical trials. SM04690 (**1**, lorecivivint) was recently reported as a highly potent (EC_50_ 20 nM), selective Wnt pathway inhibitor in a TCF/LEF reporter system in SW480 colon cancer cells.^39^ It was shown to be effective in a number of *in vitro* cellular models of osteoarthritis (OA) and efficacious in a rodent model of OA and is currently being tested in two phase 2 clinical trials for knee OA. Early reports on efficacy and safety from these trials appears promising.^40^ Samumed recently reported a novel mechanism of action for Wnt pathway inhibition in human mesenchymal stem cells for SM04690 via dual inhibition of intranuclear kinases, CLK2 (IC_50_ 8 nM) and DYRK1A (IC_50_ 30 nM).^41^ SM04755 (**2**) and SM08502 (**3**) (undisclosed structures) have also been reported as Wnt inhibitors with activity against CLK kinases and are currently in clinical trials for various disease indications.^42^ Conversely, SM04554 (**4**) is reported as a small molecule activator of Wnt signaling that has been shown to increase total and nuclear β-catenin and increase hair growth and follicle number in a depilated murine model.^43^ It is currently in phase 2/3 clinical trials as a topical agent for androgenetic alopecia.

### Targeting Wnt Signaling in the Nucleus

Targeting the machinery of the β-catenin/TCF response elements involved in regulating transcription provides a direct method for modulating Wnt-dependent gene expression. In general, drugging transcription factors is considered challenging,^44^ and although there have been reports of molecules directly binding to β-catenin,^36^ currently the most advanced strategies have targeted transcriptional coactivators. The first generation Wnt signaling inhibitor ICG-001 (**5**) was discovered through a screen with a modest 5000 compound library using a TOP-flash assay in the colon cancer SW480 cell line (IC_50_ 3 μM). Follow-up target identification studies showed that it antagonized β-catenin/TCF dependent transcription by binding the N-terminus of the transcriptional coactivator protein CBP, blocking its interaction with β-catenin while not binding the related transcriptional coactivator p300.^45^ ICG-001 was found to selectively induce apoptosis in transformed colon cells but not normal colon cells and showed efficacy in xenograft mouse models of colon cancer. Structurally related second generation phosphate prodrug PRI-724 (**6**), developed by PRISM Pharma Co., was entered into phase 1/2 clinical trials for multiple anticancer indications. Preclinical studies in mice suggested that the β-catenin/CBP interaction was important for the onset of liver fibrosis, and this was suppressed by treatment with PRI-724.^46^ Clinical trials with PRI-724 are ongoing for the treatment of liver cirrhosis. C-82 (**7**), the active form of PRI-724, entered phase 1/2 clinical trials in 2015 as a topical agent for the treatment of the skin conditions psoriasis and systemic sclerosis.^47^ CWP-291 (**8**, formerly CWP232291) is a peptidomimetic small molecule drug precursor to the active metabolite CWP232204 (structures undisclosed) developed by JW Pharmaceuticals that reduces β-catenin levels in a TOP-flash reporter assay in HEK293 cells (IC_50_ 273 nM) and is currently in phase 2 clinical trials for acute myeloid leukemia.^48^ The active form CWP-291 binds to Sam68 (an RNA-binding protein that regulates alternative splicing of the TCF-1 transcription factor in a complex with CBP) and induces β-catenin degradation.^49^

### Targeting Wnt Signaling in the Cytoplasm

The post-translational acylation of Wnt ligands is considered to be a key step prior to its secretion and activation.^11^ The endoplasmic reticulum MBOAT enzyme Porcupine specifically acylates Wnt proteins at a conserved serine residue (Ser209 in hWnt3a). Inhibition of Porcupine has been shown to impair the correct processing and secretion of Wnts resulting in decreased Wnt-dependent signaling and downstream gene expression. A number of highly potent Porcupine inhibitors have been reported, and the therapeutic potential of targeting Porcupine, particularly in oncology, has been recently reviewed elsewhere.^17,50^ Briefly, the first inhibitors of Porcupine were discovered by Chen and co-workers who utilized a chemical genetics approach to identify two distinct class of inhibitors of Wnt signaling, one class that inhibited Wnt production (IWPs) and another group that inhibited the Wnt response (IWRs) downstream of LRP6 and Dishevelled.^51^ Using biochemical markers of Wnt/β-catenin pathway activation, it was confirmed that the IWPs blocked key events across the canonical Wnt signaling cascade and follow-up target identification and validation experiments (primarily using IWP-2, **9**), demonstrated that this activity was due to specific, on-target inhibition of Porcupine. Despite IWPs’ clear value as tool molecules for probing Wnt signaling and Porcupine biology in an *in vitro* setting, their use *in vivo* has been more limited. LGK974 (**10**, WNT974), one of the first clinical candidate Porcupine inhibitors, developed by Novartis, was discovered through a ligand-based medicinal chemistry program and was shown to display exquisite potency (IC_50_ 0.4 nM) in a Wnt reporter assay in T3 cells. LGK974 also showed dose dependent efficacy as measured by tumor growth delay and regression in a Wnt-driven murine tumor model.^52^ Despite the near universal importance of Wnt acylation for Wnt signaling, LGK974 was found to be well-tolerated at the efficacious dose in both preclinical animal models and phase 1 clinical trials for solid tumors.^53,54^ Other Porcupine inhibitors to enter clinical trials include ETC-1922159, RXC004, and CGX1321. Recent identification of key binding residues for Porcupine mediated Wnt acylation^55^ and the publication of the first crystal structure of a homologous MBOAT enzyme^56^ should aid in the future identification of next generation Porcupine modulators; however management of on-target toxicity remains an ongoing challenge for Porcupine inhibitors delivered systemically.

In the case of the IWRs, Chen et al. noted that IWR-1-*endo* (**11**) inhibited Wnt signaling in a reporter assay (IC_50_ 180 nM) and showed that this occurred by increased degradation of β-catenin through increased protein levels of the key β-catenin destruction complex protein Axin.^51^ Following the identification of **11**, Huang et al. used a chemical genetic screen to identify Xav939 (**12**) that also antagonized Wnt signaling through the stabilization of Axin.^57^ It was shown that this stabilization occurred through inhibition of the poly-ADP-ribosylating (PARP) enzymes tankyrase 1 and 2 (TNKS 1 and 2), and that both IWR-1-*endo* and Xav939 were potent dual TNKS 1 and 2 inhibitors. PARylation of Axin leads to its degradation resulting in more free β-catenin in the cytosol; inhibiting PARylation reduces Axin’s turnover, which in turn increases sequestration of cytosolic β-catenin into the destruction complex. While bound in the destruction complex, β-catenin is tagged for degradation through sequential phosphorylation by CK1α and GSK3β.^58^ Since the first crystal structures of the human TNKS2 in complex with Xav939 was reported in 2009,^59^ there has been a steady stream of more potent and selective tankyrase inhibitors generated through structure-based approaches, for example, G007-LK,^60^ NVP-TNKS656,^61^ MSC2504877,^62^ and others.^35^ However, despite showing efficacy in preclinical Wnt-driven cancer models, on-target toxicity has hampered progression of tankyrase inhibitors into the clinic.^63^ Dishevelled (Dvl; 3 human orthologues) also forms part of the Wnt cytosolic machinery, and upon extracellular binding of a Wnt ligand to a FZD receptor, Dvl is recruited to the cell membrane, where it binds to the FZD intracellular C-terminal domain through a PDZ domain and to Axin through a DIX domain. Dvl binding to Axin effectively sequesters Axin out of the destruction complex resulting in increased levels of free cytosolic β-catenin and enhanced transmission of the Wnt signal.^64^ Thus, targeting Dvl provides another opportunity for influencing Wnt signaling with a small molecule. To date, most efforts have focused on Wnt inhibition by blocking the Dvl–FZD interaction through targeting the PDZ domain of Dvl. Following on from the weak hit BML-268 (**13**) against mouse Dvl-PDZ domain described by Grandy,^65^ Hori and co-workers used a combination of virtual screening and NMR titration experiments against human Dvl1 to identify five new binders of the hDvl1_PDZ_. The most potent binder, NPL-4011 (**14**) (*K*_D_ 34.5 ± 6.6 μM), a pseudo-dimeric structural analogue of **13**, was reported to bind much more strongly than **13** (*K*_D_ 954 ± 403 μM), which was rationalized, in part, to be due to structural differences between the mouse and human Dvl_PDZ_ domains.^66^ It remains to be seen whether more potent, drug-like DVL_PDZ_ inhibitors can be developed, but given the ubiquity of PDZ domains in proteins throughout the proteome, acceptable selectivity profiles will need to be carefully considered.

### Targeting Membrane Components of Wnt Signaling

There are currently no small molecules inhibitors in clinical trials that target the cell membrane bound extracellular machinery of the Wnt signaling cascade. Considering that there are 19 human Wnts and 10 FZD receptors, targeting these highly complex extracellular receptor–ligand interactions can be somewhat daunting. Functional redundancy between FZD receptors means that strategically targeting individual receptors is challenging with potential pitfalls in terms of efficacy and off-target effects. At the same time, it also offers opportunities to selectively modulate certain portions of the Wnt pathway or modulate it in a more restricted fashion, ideally in disease relevant, localized cell populations only. Such an approach may then offer advantages in terms of safety and therapeutic window compared to targeting a globally essential pathway component such as Porcupine.

Lee and co-workers used a combination of structure-based drug design (SBDD), molecular modeling, and virtual screening to construct a focused-library to screen for inhibitors against the Wnt-binding site of the extracellular FZD8 cysteine-rich domain (CRD).^67^ This approach led to a small collection of compounds, exemplified by NSC654259 (**15**), that displayed single digit micromolar activity in a Wnt 3a-dependent reporter assay in 3T3 cells. Biolayer interferometery was used to confirm binding to the FZD8_CRD_ domain (*K*_D_ 2.9 ± 2.4 μM). The authors acknowledged that the key binding residues (Leu97, Met149, Asp150) are conserved across most of the FZD receptors, suggesting that further work is required to determine their selectivity profiles. Zhao et al. recently reported that the antiepileptic drug carbamazepine (**16**) binds into a novel allosteric binding pocket of the CRD of FZD8.^68^ Initially discovered by a small molecule screen using surface plasmon resonance (SPR), the binding site of carbamazepine was definitively confirmed by X-ray crystallography. The structure revealed a well-defined mostly hydrophobic pocket but for a water bridged hydrogen bond between Tyr52 and the carboxamide of the ligand. This hydrogen bond might be important for selectivity as Tyr52 is not conserved across the FZD family. Follow-up studies using SPR showed **16** to have a *K*_D_ of 16.8 μM against FZD8_CRD_ with no apparent binding to FRZ5_CRD_ and FRZ7_CRD_, the most closely related FZD receptor CRDs. Using a modified TOP-flash reporter assay, it was shown that **16** could inhibit Wnt signaling in an engineered FZD8 HEK293 cell line, suggesting that this novel allosteric binding site could be druggable. Zhang and co-workers used the recently reported crystal structure of the transmembrane domain (TMD) of the hedgehog signaling pathway protein Smoothened, bound to a small molecule ligand,^69^ to develop a homology model for the TMD of FZD7.^70^ Through this approach they executed a structure-based virtual screen and identified a series of *N*-aryl benzimidazoles antagonists. Further design and screening led to SRI37892 (**17**), which displayed sub-micromolar activity against Wnt/β-catenin signaling in LRP-6 expressing HEK293 cells (IC_50_ = 0.78 μM) and low micromolar antiproliferation activity against the triple negative breast cancer cell lines HS578T and BT549. Most approaches for targeting FZD receptors with small molecules are in the early stages of development; however the feasibility of targeting this family of receptors has recently been enhanced by the publication of a crystal structure of the human FZD8_CRD_–Wnt3 complex.^71^

In comparison, biological approaches to targeting the membrane components of the Wnt pathway are more advanced. OMP-54F28 (**18**, ipafricept), developed by OncoMed, is a fusion protein between a truncated FZD8 receptor that contains the Wnt-binding CRD domain and the immunoglobulin IgG1 FC region.^72^ OMP-54F28 thus acts as a FZD8 decoy receptor that binds to and sequesters Wnt proteins preventing binding to endogenous FZD receptors; the net result is to functionally antagonize Wnt signaling. OMP-54F28 has completed a number of phase 1 clinical trials for various malignancies.^73^ OncoMed also has a humanized monoclonal antibody OMP-18R5 (**19**, vantictumab) in clinical trials for oncology indications. Originally designed against FZD7, it was found to bind to 4 additional FZD receptors (FZD1, 2, 5, and 8) and is thought to block Wnt signaling by direct inhibition of Wnt binding.^74,75^ Recently, Zinzalla et al. reported BI905677 (**20**) as a first-in-class potent inhibitor of Wnt signaling. BI905677 is a biparatopic antibody comprising two modules that bind unique, nonoverlapping epitopes of LRP5/6 and presumably blocks Wnt signaling by preventing formation of the FZD–Wnt–LRP5/6 trimeric complex.^76^ The extracellular secreted protein Dickkopf-1 (DKK1) competes with Wnt proteins for binding to LRP5/6 and functions as a negative regulator of Wnt signaling.^77^ Gallocyanine (**21**, NCI8642) was shown to block the LRP6–DKK1 interaction by SPR through binding to LRP6 (*K*_D_ 0.47 μM). Gallocyanine functionally restored Wnt signaling in a concentration dependent manner in a β-catenin intracellular accumulation and nuclear translocation assay, carried-out in L-cells treated with Wnt3a and DKK1 conditioned media (IC_50_ 12.6 μM).^78^ Mpousis et al. followed-up this work and have reported a series of small molecules, based on the gallocyanine core, that inhibit the LRP6–DKK1 interaction and enhance Wnt signaling with micromolar activities.^79^ It was also reported that these gallocyanine derivatives could reduce Tau phosphorylation at serine 396 in a DKK1 induced Tau phosphorylation assay in SH-SY5Y cells.^80^

### Targeting Secreted Modulators of Wnt Signaling

Secreted frizzled-related proteins (sFRPs) form a family of 5 glycoproteins that contain a CRD domain homologous to the Wnt-binding sites of FZD receptors and are generally considered to be antagonists of Wnt signaling. This antagonism has been proposed to be conferred by their CRD domain and to occur through two main mechanisms: (1) direct competition with FZD receptors for Wnt binding sites and (2) directly forming extracellular inactivating complexes with FZD receptors.^81^ Researchers at Wyeth, interested in activating Wnt signaling as a potential therapeutic mechanism for treating bone loss disorders, designed a high-throughput screen (HTS) based around an optimized TCF-luciferase reporter assay looking for activation of canonical Wnt signaling; specifically, looking for Wnt activation through sFRP-1 inhibition. Of the 685 initially confirmed hits, 65 were found to inhibit Wnt signaling in an sFRP-1 specific manner.^82^ A ligand-based drug design approach was then used to optimize hit WAY-316606 (not shown) cumulating in the potent lead compound WAY-362692 (**22**) with activity in both a fluorescent polarization binding assay (IC_50_ 20 nM) and a Wnt-activation reporter assay (EC_50_ 30 nM). Additionally, **22** was demonstrated to increase total bone area by 75% in an *ex vivo* model of bone formation.^83,84^ As discussed in the previous section, DKK1 is a secreted extracellular negative regulator of Wnt signaling. Again, researchers at Wyeth looking for Wnt agonists, used a HTS and ligand-based optimization approach to discover WAY-262611 (**23**), a small molecule inhibitor of DKK1 as measured by a TCF-luciferase reporter assay in an osteosarcoma cell line treated with Wnt3a and DKK1 conditioned media (EC_50_ 0.63 μM). WAY-262611 was optimized to be highly selective over GSK-3β, have favorable pharmacokinetic (PK) properties, and display efficacy in an *in vivo* rat model of bone formation when dosed orally.^85^

The first-in-class humanized monoclonal antibody DKN-01 (**24**), developed by Leap therapeutics, binds to and inhibits DKK1, which in turn has been shown to restore Wnt signaling. Elevated levels of circulating DKK1 has been associated with a number of neoplastic diseases, and DKN-01 is currently undergoing multiple clinical trials for various malignancies.^86^ WNT Research has developed Foxy-5 (**25**), a synthetic 6 amino acid peptide fragment of Wnt5a that mimics the effects of Wnt5a. Wnt5a is a ligand of the noncanonical Wnt signaling pathway that can also effect canonical signaling in a context and cell-type dependent manner. In certain cancers, low Wnt5a expression has been linked to increased metastasis and a poorer disease prognosis.^87^ Foxy-5 has completed phase 1 clinical trials for metastatic breast, colon, and prostate cancer and is currently recruiting for a phase 2 study for colon cancer.

Notum, a secreted carboxylesterase, has recently been identified as a negative modulator of Wnt signaling.^29^ Notum acts through the removal of an essential palmitoleoyl moiety from Wnt proteins, thereby rendering them inactive. Signaling by Wnt proteins is finely balanced and tightly regulated by a sophisticated network of modulators and feedback processes including secreted inhibitory proteins, and it is of note that Notum performs the reverse biochemical process to the intracellular enzyme Porcupine.

As an enzyme with a defined high-resolution crystal structure and druggable pocket, Notum presents a tractable target for modulating Wnt signaling. There are now a number of reports of inhibitors of Notum carboxylesterase activity providing chemical tools for use in drug target validation experiments, and a few of these inhibitors **26**–**28** have been now evaluated in PK and disease models.

## Insights from Structural Biology

Several key members of the Wnt signaling pathway have had their structures determined, allowing an enhanced understanding of their function as well as providing ways to rationally design modulators. The key signal transduction event, Wnt binding to FZD, has been captured in this manner. While the structure of the FZD CRD that serves as the Wnt binding site has been known for some time,^88^ the first structure showing Wnt binding was reported in 2012 (PDB 4F0A)^12^ and subsequent work has shown the structures also for a mammalian complex (PDB 6AHY).^71^ An interesting aspect of the Wnt–FZD complex is the direct involvement of Wnt lipidation in the binding by interaction with a hydrophobic groove present in the extracellular domain of FZD. It has long been known that post-translational addition of palmitoleate is a required feature for fully functional Wnt,^10^ but the molecular basis for the enhanced activity on the receptor is clearly illustrated by the structural information. Additional structural studies have shown that while FZD receptors display certain hallmark properties of G-protein coupled receptors (GPCRs) such as seven transmembrane regions, they are at the same time distinct from other GPCRs with large differences in the ligand binding region, possibly indicating that new approaches are required for the modulation of FZD receptors.^89^ Recently, a structure of FZD-8 bound to carbamazepine (**16**, PDB 6TFB) revealed an allosteric site that offer opportunities for future targeting of this receptor.^68^

Also, part of the mechanism for downstream signal transduction has been elucidated through the determination of various Dishevelled (Dvl) domain structures. From the first NMR structure offering a putative mechanism of signal transduction,^90^ more structures have followed including the PDZ domain with bound inhibitory peptides,^91^ the latter offering a potential approach for the development of small molecule Wnt signaling inhibitors. Additional components related to the signal transduction have also had their structures determined, including β-catenin/TCF^92^ and LRP6 in complex with DKK1.^93^

The Wnt proteins themselves are about 40 kDa in size and across the family contain a conserved serine that serves as an anchor point for a palmitoleic ester post-translational modification. Regulating the lipidation of Wnt is primarily done by the enzymes Porcupine and Notum, acylating and deacylating Wnt, respectively. No structural information is available for Porcupine, although related enzymes have been reported.^56^ However, for Notum several structures have recently been reported, offering a molecular level insight into Wnt regulation.

**Figure 4:**
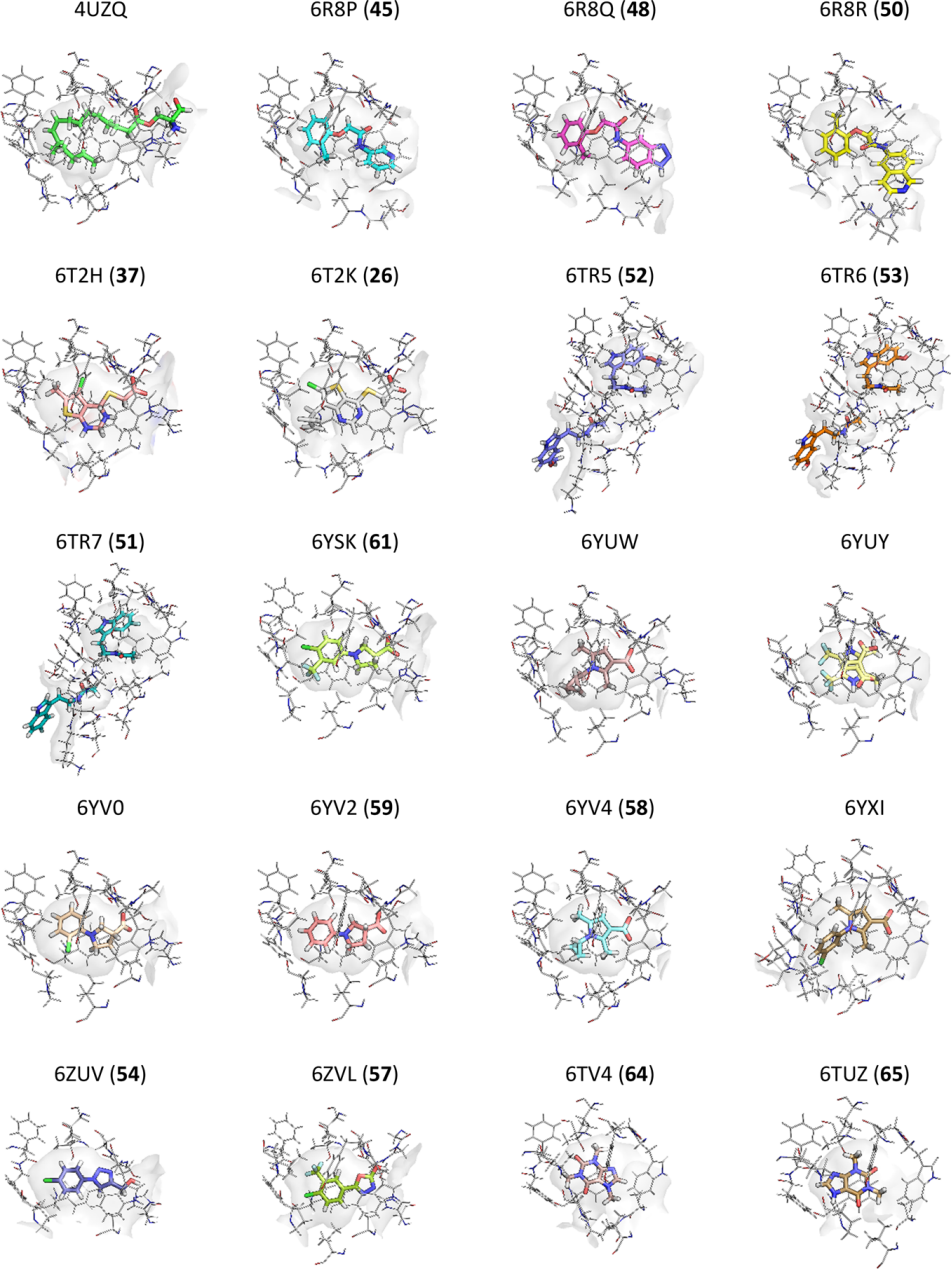
Active site of Notum structures with bound ligand published to date. Residues are shown within 4 Å of the bound ligand(s) with the pocket surface highlighted (gray). Bold number in parentheses refers to chemical structure.

The mechanism of Notum mediated hydrolysis is well understood.^29^ The basis for the ability of Notum to selectively cleave palmitoleate from Wnt is a well-defined hydrophobic pocket (∼380 Å^3^) in proximity to a typical hydrolase Ser-His-Asp catalytic triad (Ser232, His389, and Asp340 in hNotum) (Figure 2). The size of the pocket is optimal to accommodate a *cis*-unsaturated lipid at C9–C10 with a total length up to 16 carbon atoms. The structure of *O*-palmitoleoyl serine bound to Notum (PDB 4UZQ) shows the fatty acid chain occupying the pocket, positioning the ester bond in close proximity to the catalytic triad and in an ideal position for a tetrahedral transition state to be stabilized by the oxyanion hole formed by backbone amides (Gly127–Trp128, Ser232–Ala233, and Gly126–Gly127). Another feature of the Notum structure is the presence of sulfate binding sites that have been implicated in glypican binding. This binding is suggested to retain Notum at the cell surface. Several structures of drug-like molecules bound to Notum are also reported in the literature, giving valuable insight on inhibitor binding; these are discussed in more detail in later sections of this review. The available structures of Notum in the Protein Data Bank (PDB) are summarized in Table 3 and Figure 4.

**Table 3:** X-ray Crystal Structures of Notum

PDB structure codes	refs
4UZL, 4UZ7, 4UYW, 4UZA, 4UZ9, 4WBH, 4UZ1, 4UZ5, 4UYZ, 4UYU, 4UZJ, 4UZK, 4UZ6, 4UZQ	(29)
6R8P, 6R8Q, 6R8R	(123)
6TR5, 6TR6, 6TR7	(124)
6T2H, 6T2K	(117)
6YSK, 6YUW, 6YUY, 6YV0, 6YV2, 6YV4, 6YXI	(128)
6ZUV, 6ZVL	(125)
6TV4, 6TUZ	(129)

Overall, the available structural data allow us to get a fairly complete picture of the molecular mechanism of Wnt signaling and offer several exciting possibilities to leverage SBDD approaches to discover new modulators of the pathway.

## Notum and Disease

Understanding of the role of Notum in mammalian biology and disease is predicated upon the premise that loss of Notum, either through genetic manipulation or through pharmacological inhibition, leads to an increase in Wnt signaling. While certainly this is true, it remains a possibility that Notum may have other substrate(s), and therefore its manipulation may theoretically lead to changes in other extracellular signaling systems. Notum’s function is also informed by its tissue and cellular level expression: our knowledge of Notum expression and distribution remains incomplete, but at a tissue level it appears to be expressed in bladder, brain, liver, kidney, lung, skeletal muscle, and skin.^94^ As described below, more detailed studies are now defining cellular level expression.

The first clues regarding Notum function in mammals, and thereby any association with disease, came from genetically modified mice where Notum had been deleted.^95^ The Notum knockout mice were stated to have increased bone formation on endocortical (marrow-facing) bone surface, presumably due to increased local Wnt signaling. Interestingly, the knockout mice were also reported to have a defect in dentin morphogenesis, indicating a role for Notum in tooth development.^96^ The adult Notum knockout mice had slightly reduced body weight and lean body mass and body fat compared to wild-type mice, and histological analysis of 40 soft tissues revealed no phenotypic changes.^95^ The role of Notum in modulating bone was further validated using small molecule inhibitors of Notum, for example, LP-922056 (**26**), where increases in femur cortical bone thickness were reported following chronic dosing of mice.^95^ Subsequent mechanistic investigation using more sophisticated conditional and cell lineage specific Notum knockouts lead to the conclusion that osteoblast-derived Notum is a local regulator of cortical bone mass through effects on periosteal bone formation.^97^ These authors also reported that variants in the Notum gene are associated with bone density variation in adult humans. A subsequent study^98^ also described the expression of Notum in bone osteoblasts and the use of both small molecule Notum inhibitors and a neutralizing anti-Notum antibody to chronically deplete Notum activity in adult mice, leading to increased cortical bone thickness and strength. Together these reports support the concept of therapies targeting osteoblast-derived Notum to strengthen cortical bone and prevent nonvertebral fractures.

A liver specific Notum knockout mouse has also been described.^99^ Given that Wnt signaling plays a critical role in liver development, a profound phenotype may have been predicted. However, these mice did not appear to have any changes in liver development or zonation. The only phenotype reported was the development of obesity and glucose intolerance, curiously restricted to male mice. Note that this finding is in contrast to the global Notum knockout mouse.^95^ The relevance of this observation to human biology and disease is unclear.

Wnt signaling has long been recognized to play a central role in the maintenance of adult stem cell populations in tissues as diverse as the intestine, skin, and brain,^100^ particularly in the context of the so-called “stem cell niche”.^101^ Given the role of many adult stem cell populations in the ongoing regeneration and replacement of specific differentiated cell types, this leads to the notion that modulation of Wnt signaling in niche–stem cell interactions could provide an approach to therapeutically targeting tissue renewal and age-related regeneration. To date, two examples have described an important role for Notum in modulating Wnt signaling in the adult stem cell niche:

(1) In the intestine, ongoing regeneration of the epithelium is critical. This is known to be a Wnt dependent system that declines with age. Wnt signaling is essential to maintain the so-called intestinal stem cell niche, thereby enabling the ongoing generation of new epithelium. Pentinmikko et al. have found a critical role for Notum in regulating this system.^102^ It was found that Paneth cells, located in the intestinal stem cell niche, express Notum. Upon aging, the expression of Notum increases, leading to decreased Wnt signaling and subsequent reduction in stem cell maintenance and regeneration. Excitingly, the authors found that application of a small molecule Notum inhibitor, ABC99 (**27**), normalized Wnt signaling and restored epithelial regeneration. These observations open up the possibility of the therapeutic use of Notum inhibitors, for instance, to mitigate against adverse effects of chemotherapeutic agents on intestine structure and function, particularly in older patients.
(2) In the adult mammalian brain, two regions have been described where there is ongoing generation of new neurons from stem cell populations (“neurogenesis”): the subventricular zone and the subgranular zone. The existence of these in the human brain has, at times, proven controversial.^103^ Similarly, the biological role (and thereby the opportunity for therapeutic manipulation), particularly in humans, has proven difficult to investigate.^104^ As in the intestine, it is recognized that Wnt signaling is important in regulation of stem cell populations in the adult brain.^105^ Mizrak and colleagues have now discovered a role for Notum in modulating Wnt signaling, and thereby neurogenesis, in the adult mouse subventricular zone.^106^ They found that Notum, secreted by specific neural stem cell (NSC) intermediates, inhibits the proliferation of nearby NSC progeny (presumably by inhibiting Wnt signaling), thereby possibly providing a favorable environment for their cellular progeny. The downstream implications of inhibiting Notum on the migration, integration, and function of progeny neurons, and thereby any regenerative potential to treat acute and chronic neurological and neurodegenerative disease, remains to be elucidated. Additionally, whether Notum modulates neurogenesis in the other stem cell population in the brain (the subgranular zone of the hippocampus) is currently unknown.

Dysfunction of Wnt signaling is well recognized as a component of many cancer types,^107^ and indeed its modulation is considered as a valid approach for the development of targeted therapies.^108^ The role of Notum as a potential contributor to aberrant Wnt signaling in various forms of cancer is at an early stage of investigation. Nevertheless, there are some reports emerging that implicate a role. In 2008, Torisu et al. reported that Notum was overexpressed in primary hepatocellular cancer samples and that this was associated with increased nuclear β-catenin, consistent with increased intracellular Wnt signaling.^109^ The role of Notum in cancer biology or whether the upregulation of Notum represented simply a negative feedback mechanism of increased Wnt tone was not investigated. A similar upregulation of Notum was reported in tissue from an animal model of colorectal cancer and human biopsy material.^110^ Indeed, this upregulation of Notum in certain cancers has led to the suggestion that Notum levels in the plasma may be a useful biomarker of disease.^111^ An important point to consider is that the role of Notum in the development of a cancer may be analogous to its role in the adult stem cell niche described above; that is, rather than a cell autonomous role for Notum, it is possible that there is a more complex interplay between tumorigenic cells and their surrounding niche, with Notum playing a key role in aberrant Wnt signaling.

## Small Molecule Inhibitors of Notum Activity

A number of complementary strategies have been applied to discover inhibitors of Notum activity. Successful approaches that have identified drug-like small molecules include high-throughput screening (HTS) campaigns, activity-based protein profiling (ABPP), natural products, and screening of fragment libraries. Several of these inhibitors have been soaked into crystals of Notum, and the structures have been solved to determine the inhibitor binding modes (Table 3 and Figure 4). Hence, under suitable conditions, Notum is very amenable to SBDD to help guide progress toward the discovery of potent inhibitors.

**Figure 5:**
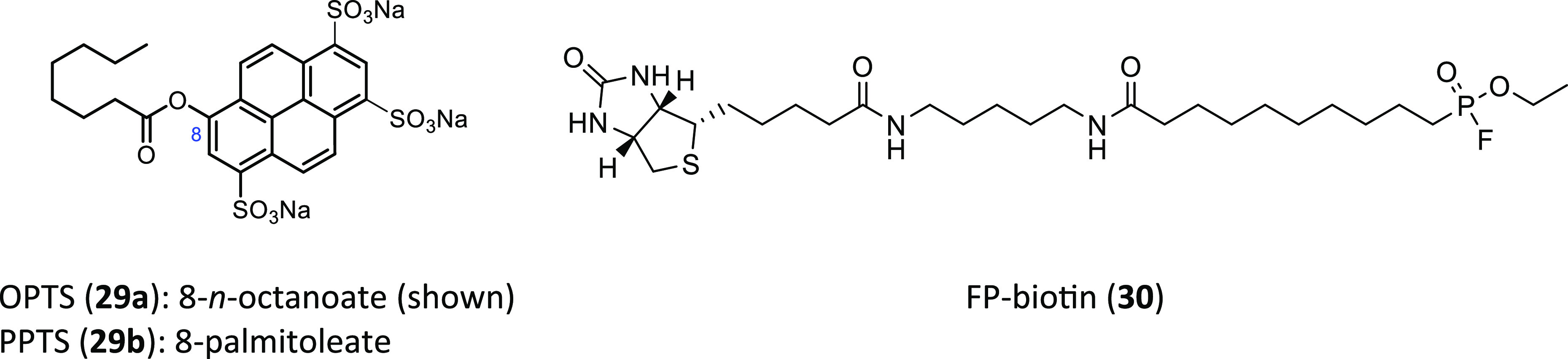
Reagents used in the identification of Notum inhibitors.

There are several screening assay formats that have been used to identify and characterize inhibitors of Notum. Inhibition of Notum carboxylesterase activity has been routinely measured in a cell-free biochemical assay with synthetic fluorescent substrates (Figure 5). Test compounds are incubated with Notum and trisodium 8-octanoyloxypyrene-1,3,6-trisulfonate (OPTS, **29a**) as the substrate, and fluorescence is recorded; an inhibitor of Notum (IC_50_) would suppress fluorescence by binding to Notum and preventing hydrolysis of OPTS. This assay can also be performed with PPTS (**29b**), which incorporates the palmitoleate group, but this requires a custom synthesis of the substrate and shorter incubation times.

Inhibitors can be screened in cell-based TCF/LEF reporter gene assays to assess their ability to restore Wnt/β-catenin signaling when activated by an exogenous recombinant Wnt in the presence of Notum. An inhibitor of Notum should show activation of Wnt signaling (EC_50_) in this model system. Performing this assay in the absence of Notum should show a maximal Wnt response at all concentrations of test compounds to confirm the outcome was due to direct inhibition of Notum activity and not related to assay interference or cell toxicity.

Inhibitors of Notum have also been tested in a Notum occupancy assay using FP-biotin (**30**),^112^ a covalent serine hydrolase activity-based probe, whereby labeling of Ser232 of Notum with FP-biotin can be blocked by an inhibitor occupying the active site of Notum. Biophysical screening methods, such as differential scanning fluorimetry (DSF) (thermal shift assay, Δ*T*_m_) and SPR, can provide additional information on affinity and binding kinetics.

### Notum Inhibitors Identified by High-Throughput Screening

The first report of small molecule inhibitors of Notum activity in 2012 came from Lexicon Pharmaceuticals, Inc. (US) as patent applications based upon fused heterocyclic scaffolds.^113,114^ These initial patent disclosures gave some understanding of structure–activity relationships (SAR), which would be expanded upon in detail in later publications.

**Figure 6:**
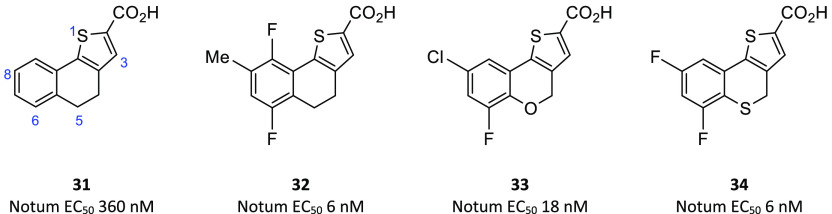
Tricyclic heterocyclic acids as first generation Notum inhibitors from Lexicon Pharmaceuticals, Inc. IC_50_ and EC_50_ values presented in Figures 6–20 refer to the Notum OPTS and TCF/LEF assays, respectively, unless stated otherwise.

Using a HTS campaign with a TCF/LEF Cell sensor reporter assay, Han et al. identified a range of fused heterocyclic compounds as potent inhibitors of Notum (Figure 6).^115^ The initial lead carboxylic acid **31** was selected for further development with modifications of the carboxyl group, the tricyclic core and substitution of the aromatic rings. Interestingly, while secondary and tertiary carboxamides were tolerated, primary amides showed significant improvements in potency over the parent acid. However, profiling of these amides in liver microsomes showed them to have poor metabolic stability, and so amides were not investigated further. SARs then focused upon retaining the carboxylic acid and exploring the tricyclic motif.

The S1 atom of the thiophene ring was shown to be crucial for activity, as was the sp^2^ C3 atom, and neither could be replaced. The importance of the distal phenyl ring was also demonstrated, with analogues bearing heteroaromatics having greatly reduced activities. The 5-position of the dihydronaptho ring was shown to be tolerant of changes in heteroatom, with potency activity in the order of S > C > O. A thorough SAR investigation of substitutions on the distal phenyl ring of the dihydronaptho analogue **31** showed that a range of substitution patterns and substituents were tolerated and superior to R^6^–R^9^ = H. The most active compound in this set was **32**, which gave potent inhibition of both human and mouse Notum. The corresponding chromene analogues, for example, **33**, were generally less potent, although similar SAR trends were observed.

The effect of substitution on the phenyl ring of the thiochromene core also followed the same general trend with multiple halogen substituents generally improving potency, which led to the development of the 6-F,8-F analogue **34**. Compound **34** was evaluated in mouse PK studies and, when dosed orally (10 mg/kg), showed good exposure (*C*_max_ 16 μM; AUC 56 μM·h) and moderate clearance (Cl 19 mL/min/kg). Compound **34** was then advanced into a mouse model of bone growth where F1 male hybrid (129xC57) mice were treated for 28 days with compound administration in their diet (5 and 20 mg/kg). Mice treated with **34** showed dose-dependent increases in midshaft femur cortical thickness of 4% and 8% over control.

**Figure 7:**
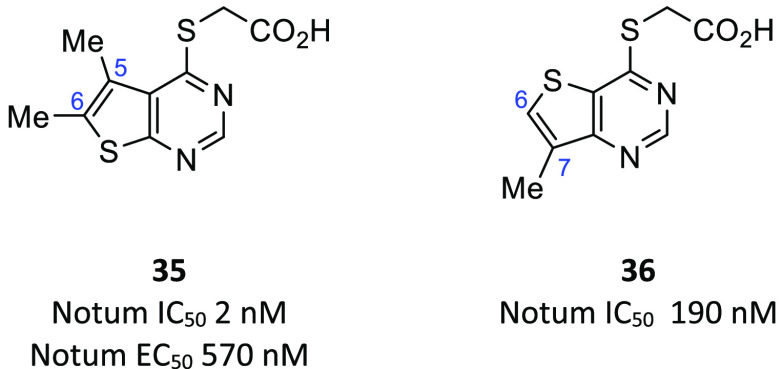
Lexicon [2,3-*d*]pyrimidine acid **35** HTS hit and isomer [3,2-*d*]pyrimidine acid **36**.

Lexicon then published a second lead series from their HTS with [2,3-*d*]pyrimidine acid **35** as a hit that showed activity in both the OPTS and TCF/LEF assays, which offered a good starting point for optimization (Figure 7).^95^ A range of alternative 5,6-heterocycles were synthesized and evaluated, with significant loss in potency. The only tolerated change was transposition of the thiophene ring to give the isomeric thieno[3,2-*d*]pyrimidine **36**, and so **35** and **36** were selected for further development.

**Figure 8:**
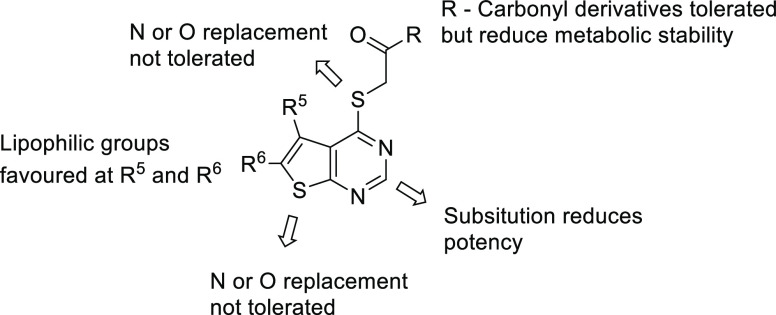
General SAR trends of the [2,3-*d*]pyrimidine acid template **35**.

**Figure 9:**
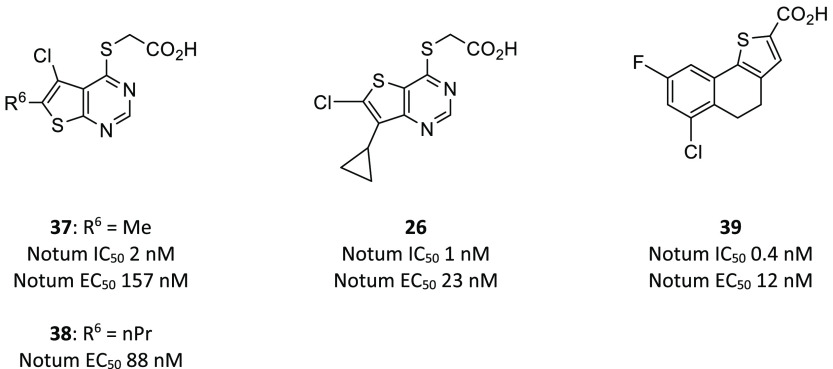
Lexicon optimized leads LP-914822 (**37**), LP-922056 (**26**), and LP-935001 (**39**).

A systematic investigation in the [2,3-*d*]pyrimidine acid series **35** at the 5- and 6-positions showed that Notum inhibition could be improved to EC_50_ ca. 100 nM by suitable choice of substituents with R^5^ = Cl and R^6^ = Me (**37**) or nPr (**38**) being the most potent from this set (Figures 8 and 9). The acid of the original lead **35** was converted to a number of carbonyl derivatives (esters, amides, ketones, and *N*-acylsulfonamides) but these modifications offered no advantage, and the carboxamides again suffered from poor metabolic stability. In contrast, the parent carboxylic acids had good ADME properties.

The thieno[3,2-*d*]pyrimdine scaffold **36** was explored in a similar manner to **35**, which identified **26** as one of the most potent inhibitors prepared to date (Figure 9). Compound **26** was evaluated in mouse PK studies (10 mg/kg po) and showed high exposure (*C*_max_ 129 μM; AUC 1533 μM·h) and low clearance (Cl 0.49 mL/min/kg) that was superior to thiochromene **34**. Compound **26** was also assessed in the same mouse model of bone growth, and mice treated daily with **26** (3, 10, 30 mg/kg, po) for 25 days showed increases in midshaft femur cortical thickness at all doses tested.

LP-914822 (**37**), LP-922056 (**26**), and LP-935001 (**39**) (Figure 9) emerged as three advanced leads for the program and were used to demonstrate in rodent pharmacology studies, along with complementary approaches, that inhibition of Notum activity is a potential novel anabolic therapy for strengthening cortical bone and preventing nonvertebral fractures.^98^

Additional mouse PK data for **26** was generated to evaluate brain penetration.^116^ Following a single oral dose (10 mg/kg, po), the plasma parameters from these experiments (*C*_max_, AUC, and *t*_1/2_) were consistent with published data.^95^ Brain penetration of **26** is very low with brain/plasma concentration ratios ∼0.01 at all time points measured up to 24 h and also 0.01 based on AUC_(0→inf)_. Hence, **26** is unsuitable for use in models of disease where brain penetration is an essential requirement.

### Scaffold-Hopping Approaches

**Figure 10:**
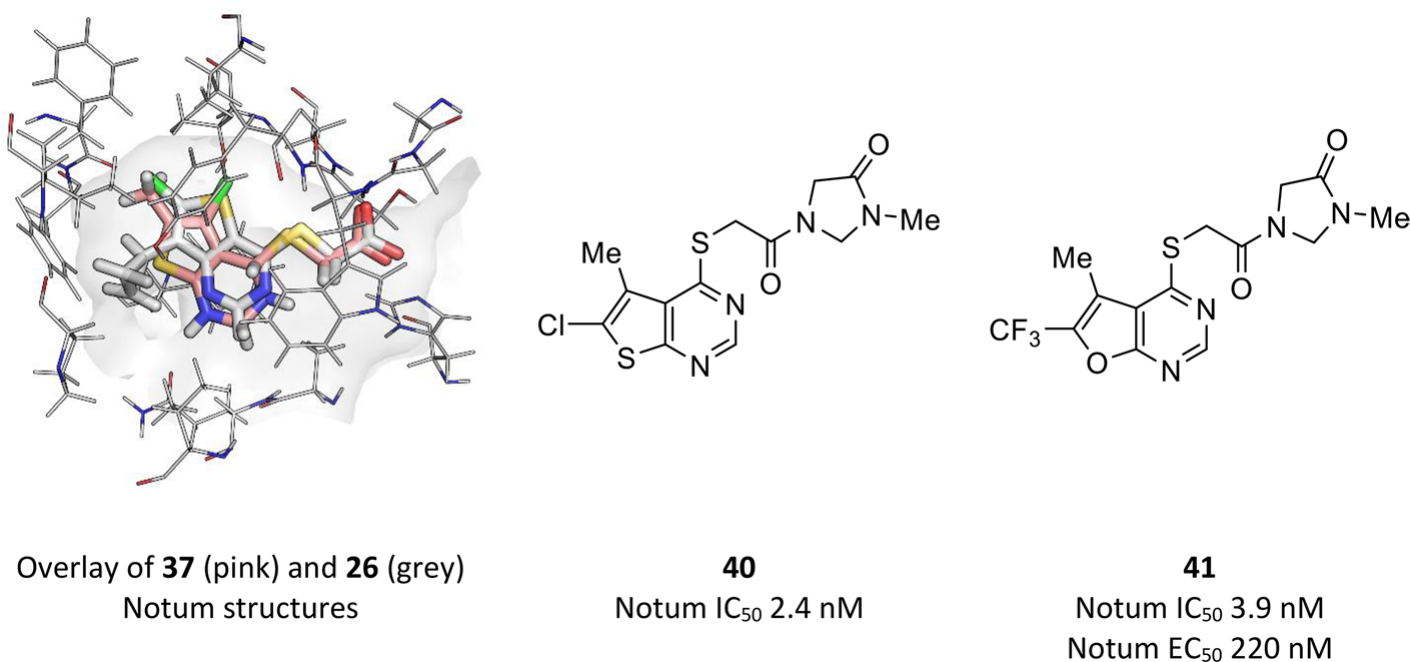
Scaffold-hopping, supported by X-ray structure determination, identified furano[2,3-*d*]pyrimidine amide **41** with reasonable brain permeability.

The thienopyrimdine scaffolds **37** and **26** were revisited by Atkinson et al. at University College London (UCL) in an attempt to introduce brain permeability.^117^ A small library of diverse carboxamides was prepared from acids **37** and **26** to explore if they could be modified to deliver a central nervous system (CNS) penetrant tool by capping off the acid as an amide. This strategy was guided by SBDD with the determination of Notum structures with **37** (PDB 6T2H) and **26** (PDB 6T2K) (Figure 10). Both **37** and **26** place the thienopyrimidine group in the palmitoleate pocket but with the thiophene ring at a slightly different position to accommodate the substituents; the remainder of these templates adopt a similar position. From a design perspective, these structures show significant space at the mouth of the pocket to accommodate a suitable group as an amide derivative of **37** or **26**.

Although significant Notum inhibition activity could be achieved (IC_50_ < 10 nM), none of these amides demonstrated the required combination of metabolic stability along with cell permeability without evidence of P-glycoprotein (P-gp) efflux to varying degrees. The most advanced compound from this set, **40**, was assessed in mouse PK (10 mg/kg, po) and showed low plasma exposure, which was attributed to high clearance and highlighted the need to further improve metabolic stability.

Scaffold-hopping from thienopyrimdine **40** identified furano[2,3-*d*]pyrimidine amide **41** as a potent inhibitor of Notum with improved stability in mouse liver microsomes (MLM). Crucially, it was found that by combining the furano[2,3-*d*]pyrimidine heterocycle with the preferred *N*-methylimidazolidin-4-one amide gave **41** with the best *in vitro* ADME profile of the compounds tested. The lower lipophilicity of **41** (clogP 2.1, logD_7.4_ 1.6) compared to **40** (ΔclogP = −0.5) offered an explanation for the higher microsomal stability (MLM, Cl_i_ 6.9 v 25 μL/min/mg protein, respectively). In addition, **41** was stable in mouse plasma and did not inhibit CYP450 enzymes. Compound **41** had a modest efflux ratio (ER = 2.4) in the MDCK–MDR1 permeability assay suggesting some recognition by P-gp mediated efflux transport.

PK experiments with **41** were performed in mouse with a single oral dose (10 mg/kg, po) to determine plasma exposure and brain penetration. The plasma half-life was modest (*t*_1/2_ 0.6 h), which was somewhat unexpected based on the *in vitro* MLM and plasma stability data. Brain penetration was reasonable with a brain/plasma concentration ratio of 0.29. The incomplete brain penetration was probably due to P-gp mediated efflux as shown in the asymmetry in the MDCK-MDR1 permeability assay.

### Covalent Inhibitors Identified by Activity-Based Protein Profiling (ABPP)

In 2018 Cravatt et al. described the development of a series of potent and selective irreversible Notum inhibitors discovered using gel-based activity-based protein profiling (ABPP).^118^ ABPP is a chemical proteomics approach that uses chemical probes to investigate the functional state of enzymes directly in native systems. ABPP probes have been developed that react selectively with most members of specific enzyme classes such as serine hydrolases (SH).^119,120^

**Figure 11:**
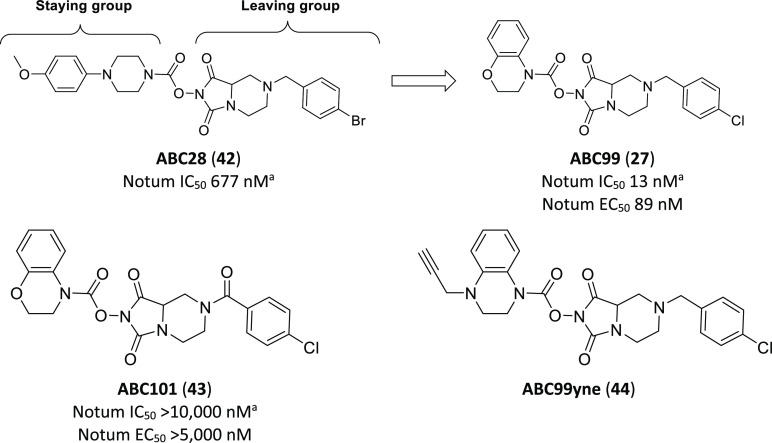
Irreversible inhibitor of Notum activity, **27**, along with structure-matched inactive control **43** and clickable probe **44**. ^a^Notum IC_50_ determined by competitive gel-based ABPP.

Having established that Notum activity could be assayed with SH-directed fluorophosphonate (FP) ABPP probes, Cravatt et al. screened a diverse set of inhibitors containing triazole urea and *N*-hydroxyhydantion (NHH) carbamate reactive groups. A series of NHH carbamates, exemplified by ABC28 (**42**) (Figure 11), were taken forward for optimization, due to their comparatively modest serine hydrolase promiscuity. Benzomorpholine replacement of the 4-phenylpiperidine “staying group” and SAR investigation of the phenyl ring resulting in a direct chlorine replacement of the bromine moiety provided significant improvements in selectivity and potency to give ABC99 (**27**). During the course of developing ABC99, it was discovered that oxidation of the benzylic position provided amide ABC101 (**43**) as a useful inactive control. In addition to gel-based ABPP, the selectivity of ABC99 was characterized using quantitative mass spectrometry (MS)-based ABPP in conditioned media and SW620 *in situ* treated cells. These data showed that ABC99 was highly selective for Notum over the 64 other serine hydrolases quantified.

The authors were interested to use these probe compounds in order to evaluate the activity of Notum in native biological systems and, for that purpose, designed ABC99yne (**44**) as a clickable probe, which incorporated an alkyne into the staying group. ABC99yne exhibited a sub-micromolar IC_50_ value and highly selective labeling of Notum in the conditioned media of SW620 cells, when samples were treated with a rhodamine-azide reporter tag under copper mediated click conditions.

ABC99 has been used to investigate the role of Notum produced by Paneth cells in regulating the intestinal stem cell niche of the small intestine.^102^ As aged Paneth cells exhibit significant Notum overexpression, it was hypothesized that increased Notum secretion was inhibiting ISC Wnt signaling and ultimately reducing intestinal regeneration. Application of ABC99 normalized Wnt signaling and restored epithelial regeneration.

### Screening of the XChem Diversity Fragment Library

In order to identify new small molecule inhibitors of Notum, Zhao and Jones performed a crystallographic fragment screen using the XChem platform at Diamond Light Source (Oxford, UK).^121^ Crystals of C-terminal His-tagged Notum (Ser81-Thr451 Cys330Ser) were soaked with the DSi-Poised library (XChem, 768 fragments).^122^ Notum has a well-defined, large, hydrophobic active-site pocket adjacent to the catalytic triad that accommodates the palmitoleate group of Wnt, and the 60 fragments observed to bind in this pocket were all resupplied as solid samples by compound purchase or resynthesis. Inhibition of Notum carboxylesterase activity of these hits was then measured in a biochemical assay. To date, three of these hits have been disclosed along with descriptions of their optimization to more potent inhibitors.^123−125^

#### 2-Phenoxyacetamides

**Figure 12:**
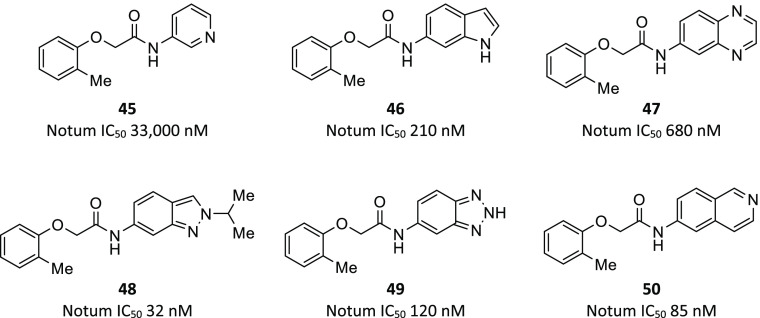
Optimization of 2-phenoxyacetamide fragment hit **45** through modification of the *N*-acetamide identified **46**–**50**.

**Figure 13:**
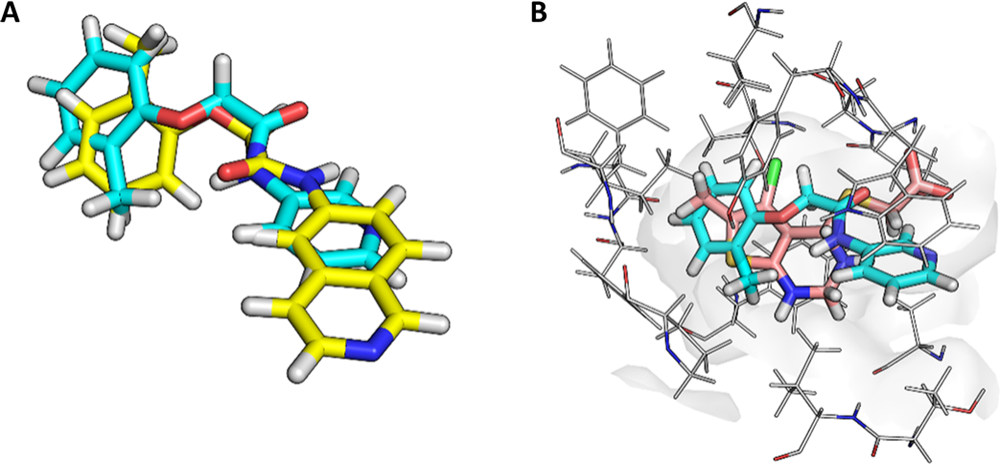
(A) Overlay of **45** (teal) and **50** (yellow). Isoquinoline **50** shows a flipped binding mode when compared to **45** and **49**. (B) Overlay of **37** (pink) and **45** (teal) Notum structures showing the different orientations of these templates.

Using this XChem screening platform, Atkinson et al. developed a series of 2-phenoxyacetamides as inhibitors of Notum activity.^123^ The fragment hit 2-(2-methylphenoxy)-*N*-(pyridine-3-yl)acetamide (**45**; IC_50_ 33 μM) was selected for further investigation for several reasons including excellent hit-like properties, synthetic tractability to explore SAR, and the Notum–**45** structure to guide compound design (Figures 12 and 13). The SAR studies systematically explored each structural feature of the inhibitor and most changes were found to be detrimental to activity. There was limited scope to modify the substituents on the phenoxy ring with small lipophilic groups in the 2-position preferred (2-Cl; 2-CF_3_) but offering minimal improvement over **45** (2-Me). These results were consistent with the structural information showing **45** (PDB 6R8P) sitting deep in the palmitoleate pocket with minimal space to accommodate a group larger than H or F at the 3- or 4-positions. In contrast, modification of the *N*-acetamide group gave the most potent inhibitors in this series. Large increases in potency over **45** could be achieved by replacement of the original 3-pyridine with benzo-fused heterocycles such as indole **46** and quinoxaline **47**. Further investigation of these 6,5- and 6,6-ring systems achieved gains in potency with indazole **48** and isoquinoline **50** as the most potent inhibitors in this series.

Structural studies with **49** (PDB 6R8Q) and **50** (PDB 6R8R) showed some unexpected features in their binding modes with Notum despite their similar potencies. Both **49** and **50** form π–π stacking with Trp128 through their heterocyclic rings, but there was a marked change in the orientation of their phenoxyacetamide backbones. Benzotriazole **49** binds in a similar mode to fragment **45**, whereas **50** shows a rotation of 180° around the amide bond, positioning the methyl of the tolyl group on the opposite side of the pocket and the carbonyl oxygen pointing away from the oxyanion hole.

Progress in this series was limited to improving potency of fragment hit **45** into **48** (1000-fold increase in activity) because it was not possible to combine Notum inhibition activity with metabolic stability in liver microsomes. All examples from this 2-phenoxyacetamide series screened in HLM and MLM were rapidly metabolized in an NADPH-independent manner with short half-lives (*t*_1/2_ < 12 min) limiting their use to *in vitro* models.

#### Melatonin and Related Structures

**Figure 14:**
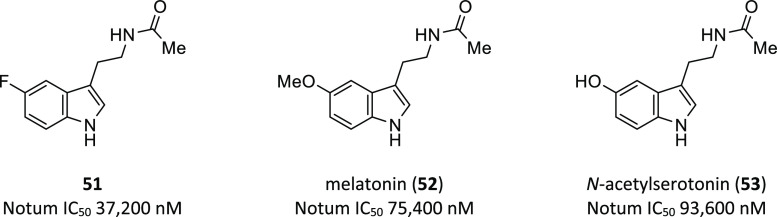
Notum inhibitors related to the hormone melatonin.

The XChem platform output was explored by Zhao et al. to identify inhibitors of Notum, and fragment hit *N*-[2-(5-fluoro-1*H*-indol-3-yl)ethyl]acetamide (**51**; IC_50_ 37.2 μM; PDB 6TR7) was highlighted due to its structural similarity to the brain hormone melatonin (**52**) (Figure 14).^124^ Additional structural studies were performed with **52** (PDB 6TR5) and *N*-acetylserotonin (**53**; PDB 6TR6) by soaking into Notum crystals, and high-resolution structures of their complexes were obtained. In each of these structures, two molecules bind with Notum: one at the enzyme’s catalytic pocket and the other at the edge of the pocket opposite the substrate entrance.

The structural information reported may guide the design new of potent inhibitors of Notum although it will be essential to develop selectivity over the melatonin receptors (MT_1–3_).

#### Development of 5-Phenyl-1,3,4-oxadiazol-2(3*H*)-ones and 1-Phenyl-1,2,3-triazoles

**Figure 15:**
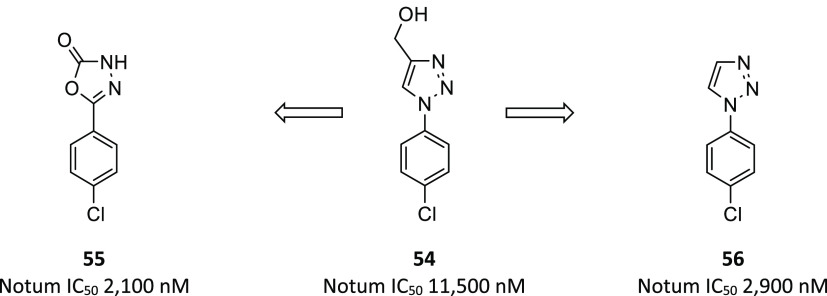
Fragment hit **54** yielded two complementary leads in oxadiazole **55** and triazole **56**.

A standout hit from this fragment set was (1-(4-chlorophenyl)-1*H*-1,2,3-triazol-4-yl)methanol (**54**; IC_50_ 11.5 μM), which was selected as a starting point for a hit-to-lead program (Figure 15).^125^ The X-ray structure of **54** (PDB 6ZUV) showed occupation of the palmitoleate pocket by the 4-chlorophenyl ring, forming a π–π stacking interaction with residue Phe268. The triazole headgroup shows possible π–π stacking interactions with Trp128 and hydrogen bonding between N2 of the triazole and peptidic backbone of Trp128. Residue Trp128 is also involved in a hydrogen bond to the oxygen of the methyl alcohol group. Optimization of **54** by modification of the heterocyclic headgroup identified two complementary leads: oxadiazole **55** and triazole **56**.

**Figure 16:**
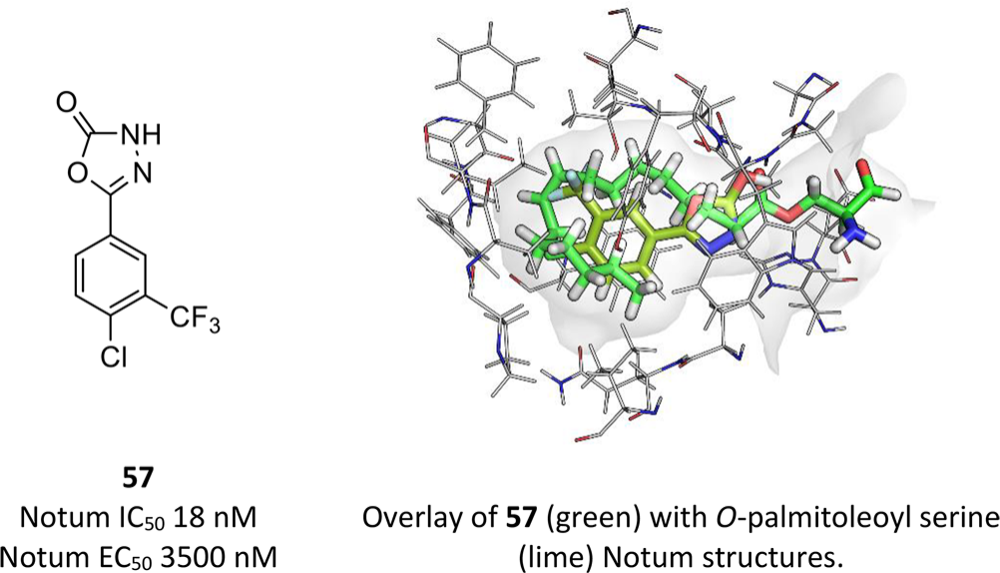
Optimized 1,3,4-oxadiazol-2(3*H*)-one **57** forms H-bonds with the oxyanion hole and fills the palmitoleate pocket.

Further investigation of the oxadiazole series **55** by exploring substitution on the aryl ring, which binds deep in the palmitoleate pocket, identified **57** as a preferred example from the 37 analogues synthesized (Figure 16). Compound **57** has physicochemical properties consistent with drug-like chemical space and contains a weakly acidic proton with a measured p*K*_a_ = 6.7. Compound **57** demonstrated good metabolic stability and cell permeability with no evidence for P-gp mediated efflux. Compound **57** restored Wnt/β-catenin signaling in a cell-based TCF/LEF reporter gene assay and prevented labeling by FP-biotin, confirming competitive binding to Notum. PK studies with **57** were performed *in vivo* in mouse with single oral administration of **57** showing good plasma exposure and partial brain penetration (brain/plasma ratio, 0.16).

An X-ray crystallographic structure determined the binding mode of **57** (PDB 6ZVL) in Notum. Inhibitor **57** makes an effective interaction with the oxyanion hole with hydrogen bonds to three separate amino acids (Gly127, Trp128, Ala223) while still filling the hydrophobic palmitoleate pocket (Figure 16).

**Figure 17:**
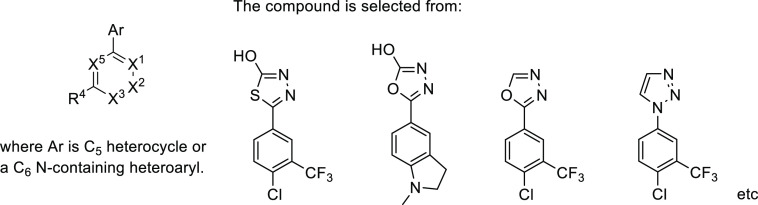
Markush structure and representative preferred compounds (4 out of 15 shown) from patent application WO 2020043866.

Significant progress was made in developing X-ray fragment hit **54** into lead **57** (>600-fold increase in activity), which showed good plasma exposure in mouse PK experiments. Furthermore, contemporaneous studies with alternative lead triazole **56** identified ARUK3001185 (**28**, only Markush structure disclosed), which was shown to be a potent, selective, and brain penetrant inhibitor of Notum activity suitable for use in both cellular and *in vivo* models of CNS disease (Figure 17).^125−127^

### Custom-Designed Fragment Libraries

In parallel to the application of the XChem platform to find fragment hits, Mahy et al. designed and screened a second library to identify alternative chemical scaffolds as inhibitors of Notum.^128^ Whereas the original DSi-Poised fragment library was a diversity subset (768 compounds), this second library was a custom-designed fragment library of carboxylic acids (250 compounds) where each member incorporated the following: a carboxylic acid to interact with the catalytic triad; a lipophilic group to be presented into the palmitoleate pocket; and a linker of various lengths to connect the acid to the lipophilic group.

**Figure 18:**
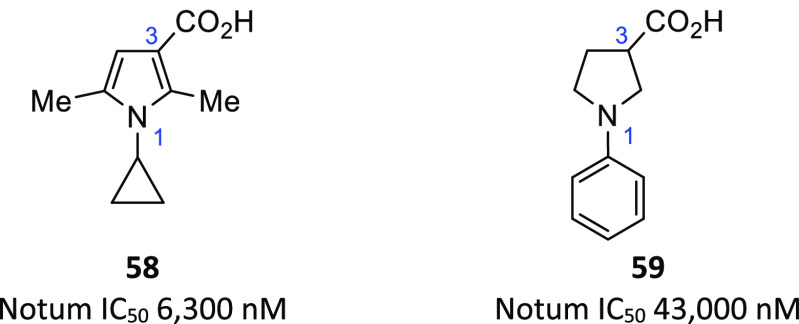
Representative hits **58** and **59** from a custom-designed acid fragment library.

Inhibition of Notum activity of this library was measured in a biochemical assay with OPTS as the substrate, and 20 compounds were identified as fragment hits (IC_50_ < 25 μM). All 20 hits were soaked into crystals of Notum, and X-ray structure determination showed that 14 fragments bound in the palmitoleate pocket. From this set, two preferred hit series were selected for further optimization: pyrrole-3-carboxylic acids (e.g., **58**; PDB 6YV4) and pyrrolidine-3-carboxylic acids (e.g., **59**; PDB 6YV2) (Figure 18).

**Figure 19:**
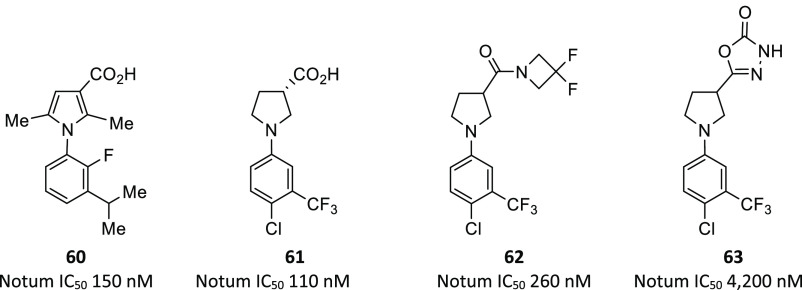
Notum inhibitors pyrrole **60** and pyrrolidines **61**–**63** derived from fragment hits **58** and **59**, respectively.

Optimization of pyrrole **58** by SAR studies guided by SBDD identified 1-phenylpyrrole **60** (clogP 5.5) as the most potent compound from this series, albeit with relatively high lipophilicity (Figure 19). Focus then switched to the pyrrolidine series **59** as this template offered the advantage of significantly lower lipophilicity when compared to the matched pyrrole but with weaker activity. Optimization of 1-phenylpyrrolidine **59** gave acid (*S*)-**61**, amide **62**, and oxadiazolone **63**. Once again, the 4-chloro-3-(trifluoromethyl)phenyl group was preferred (cf. **57**) and a Notum–**61** (PDB 6YSK) structure showed that the inhibitor fully occupied the palmitoleate pocket while making effective interactions with the oxyanion hole.

This set of inhibitors, **60**–**63**, were assessed in *in vitro* ADME assays to compare their aqueous solubility, microsomal stability, and cell permeability. These inhibitors were selected based on their Notum activity but also their chemical structural diversity and complementary physicochemical properties (clogP, p*K*_a_). On balance, pyrrolidine-3-acid **61** demonstrated a superior profile. The design and screening of a second fragment library further confirms that inhibition of Notum activity can be achieved by small, drug-like molecules possessing favorable *in vitro* ADME profiles.

### Natural Products

**Figure 20:**
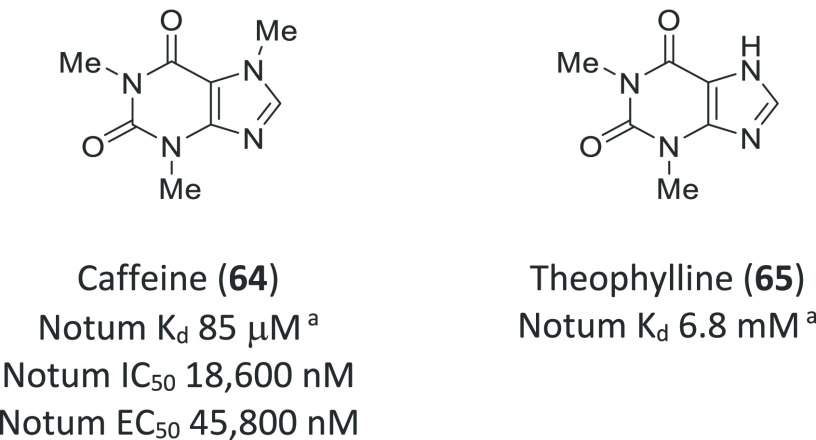
Caffeine (**64**) and theophylline (**65**) are inhibitors of Notum activity. ^a^Determined by surface plasmon resonance (SPR).

Notum activity can be inhibited by caffeine (**64**) and, to a lesser degree, by theophylline (**65**) (Figure 20).^129^ The caffeine–Notum interaction was thoroughly characterized by both biochemical and biophysical methods. High-resolution structures of **64** (PDB 6TV4) and **65** (PDB 6TUZ) show that both compounds bind at the center of the palmitoleate pocket but with quite different binding modes. This structural information may guide the design of more potent Notum inhibitors.

## Future Perspectives and Conclusion

Regulation of the Wnt signaling pathway is critically important for a number of cellular processes in both development and adult mammalian biology. Conversely, dysregulation of Wnt signaling can disrupt normal development and has been associated with a number of human diseases. The Wnt pathway has attracted significant interest for the development of new drugs, although it is proving to be challenging to identify new chemical entities or biologics that have progressed to advanced clinical trials. Modulation of the Wnt pathway requires careful selection of the molecular target to give therapeutic benefit in disease without safety issues linked to the target and, possibly, pathway. In addition, some of these targets would not be defined as “druggable” with a clearly defined site or pocket for binding a small molecule. Despite this, a number of potential drugs in the Wnt pathway have now progressed beyond human PK and safety studies, and evaluation of their potential benefit in treating disease (cancer and others) is ongoing in a number of phase 2 and 3 clinical trials.

Recently, Notum was shown to act as a negative regulator of Wnt signaling through the removal of an essential palmitoleate group; this group is required for binding of the Wnt proteins to the FZD receptors. Hence, inhibition of Notum carboxylesterase activity may represent a new approach to treat disease where dysregulation of Wnt signaling is an underlying cause and Notum has been identified as the source.

Notum is emerging as a druggable target to modulate Wnt signaling. A number of reliable screening technologies are available to identify inhibitors of Notum and these have been successfully combined with complementary screening strategies (HTS, ABPP, fragment libraries) to identify hits. Structural biology is also making an important contribution as several of these hits have been soaked into crystals of Notum and the structures have been solved to determine their binding modes. Hence, Notum can be very amenable to SBDD to help guide progress toward the discovery of potent inhibitors. A selection of these hits have been optimized to give fit-for-purpose small molecule inhibitors of Notum.

Three noteworthy examples are LP-922056 (**26**), ABC99 (**27**), and ARUK3001185 (**28**) (Table 4), which are valuable chemical tools for exploring the role of Notum in Wnt signaling at a cellular level and for use in animal disease models, that is, target validation experiments. These chemical tools have complementary profiles, and we suggest that they should be properly aligned for application in animal models. All three have been used successfully to demonstrate modulation of function in cellular and animal models at reasonable doses. Acid **26** has excellent plasma exposure upon oral dosing but very high plasma protein binding in mouse that will reduce free drug levels. In addition, **26** has negligible brain penetration in wild-type mouse. *N*-Hydroxyhydantion carbamate **27** is an irreversible inhibitor of Notum and has been dosed chronically by intraperitoneal injection although no drug levels were reported. ARUK3001185 (**28**) is a potent, selective, and brain penetrant inhibitor of Notum activity suitable for use in both cellular and *in vivo* models of CNS disease. However, available reports on the properties of **28** are currently limited to the patent literature and presentations at conferences.

**Table 4:** Summary of Properties of Notum Inhibitors LP-922056 (**26**), ABC99 (**27**), and ARUK3001185 (**28**)

	LP-922056 (**26**)	ABC99 (**27**)	ARUK3001185 (**28**)
*Physicochemical Properties*			
mol wt	300	456	281
clogP	3.1	4.3	3.9
logD_7.4_	nd^a^	nd	1.3
*Notum Inhibition*			
OPTS, IC_50_ (nM)	1.1	170^b^,^c^	6.5
gel-based ABPP, IC_50_ (nM)	nd	13	nd
TCF-LEF, EC_50_ (nM)	23	89	110
*Selectivity*			
serine hydrolases (number screened)	nd	yes (64)	yes (49)
drug targets (number screened)	nd	nd	yes (47)
kinases (number screened)	nd	nd	yes (485)
*Mouse Pharmacokinetics (1 mg/kg iv and 10 mg/kg po)*			
half-life (*t*_1/2_, h)	8.3	nd	2.4
oral bioavailability (*F*_o_, %)	65	nd	68
exposure (*C*_max_) (po)	129 μM	nd	2300 ng/mL^d^
exposure (AUC_∞_) (po)	1533 μM*h	nd	10 800 (ng·h)/mL^d^
mouse plasma protein binding (mPPB) (*f*_u_, %)	0.1	nd	4.2
brain/plasma ratio (*K*_p_) (po)	<0.01	nd^e^	1.08
*Mouse In Vivo Studies*			
route of administration and dosing regime	3, 10, 30 mg/kg, po, 25 days	10 mg/kg, ip, 7 days	2 × 30 mg/kg bid, po, 30 days
*Rat In Vivo Studies*			
route of administration and dosing regime	30 mg/kg, po, 126 days	nd	nd
refs	(95, 98, 116)	(102, 106, 118)	(125−127)

a nd, not determined or not disclosed.

b Notum IC_50_ data presented for comparison in a common assay format.

c As a covalent inhibitor, the IC_50_ value will be time dependent.

d For ease of comparison of *C*_max_ and AUC_∞_ data, 2300 ng/mL is equivalent to 8.2 μM and 10 800 (ng·h)/mL to 38 μM*h, respectively.

e ABC99 is reported to be brain penetrant in mouse; see, ref (106).

For a molecular target to be druggable, it will need to show an appropriate safety window (therapeutic index) in the patient population. As small molecule inhibitors of Notum activity are still relatively early in the drug discovery pipeline (preclinical), a preliminary drug safety assessment of modulating Notum has to be based on available mouse genetic knockout data and *in vivo* rodent toleration information.^95−99^ These studies do not raise any significant safety issues at this time. Ultimately, the safety of inhibiting Notum will need to be tested in toxicology studies where any on-target effects have been disconnected from any compound related toxicity.

Looking ahead, based on current precedent, it seems feasible to generate additional inhibitors of Notum activity. These will be of most value if they have significantly improved profiles (activity, selectivity, PK) or from alternative chemotypes with a different risk–benefit profile. Fragment screening seems to have been a successful strategy to discover new hits, although this growing body of screening data, structural information and chemical structures will facilitate virtual screening (VS) and artificial intelligence (AI) design approaches as well.

As these first generation inhibitors of Notum show utility in disease models and are then progressed to safety studies, it will possible to determine if Notum proves to be an attractive molecular target in modulating Wnt signaling that will ultimately deliver new drug candidates for use in clinical studies.

## Abbreviations Used

ABPP: activity-based protein profiling
ADME: absorption, distribution, metabolism, and elimination
BBB: blood–brain barrier
CNS: central nervous system
CRD: cysteine-rich domain
DKK1: Dickkopf
Dsh: Dishevelled (*Drosophila*)
Dvl: Dishevelled (mammalian)
ER: efflux ratio
FP: fluorophosphonate
FZD: Frizzled
HLM: human liver microsomes
HTS: high-throughput screen
LRP5: low-density lipoprotein receptor-related protein 5
MLM: mouse liver microsomes
OPTS: trisodium 8-octanoyloxypyrene-1,3,6-trisulfonate
PDB: Protein Data Bank
P-gp: P-glycoprotein
PD: pharmacodynamics
PK: pharmacokinetic
SAR: structure–activity relationship
SBDD: structure-based drug design
SPR: surface plasmon resonance
TMD: transmembrane domain
